# Cutting the Flesh: Surgery, Autopsy and Cannibalism in the Belgian Congo

**DOI:** 10.1017/mdh.2017.5

**Published:** 2017-04

**Authors:** Sokhieng Au

**Affiliations:** Department of History, KU Leuven, Blijde-Inkomststraat 21 – Box 3307, 3000 Leuven, Belgium

**Keywords:** Belgian Congo, Colonial medicine, Surgery, Anatomy, Witchcraft, Autopsy

## Abstract

Within the colonial setting of the Belgian Congo, the process of cutting the body, whether living or dead, lent itself to conflation with cannibalism and other fantastic consumption stories by both Congolese and Belgian observers. In part this was due to the instability of the meaning of the human body and the human corpse in the colonial setting. This essay maps out different views of the cadaver and personhood through medical technologies of opening the body in the Belgian Congo. The attempt to impose a specific reading of the human body on the Congolese populations through anatomy and related Western medical disciplines was unsuccessful. Ultimately, practices such as surgery and autopsy were reinterpreted and reshaped in the colonial context, as were the definitions of social and medical death. By examining the conflicts that arose around medical technologies of cutting human flesh, this essay traces multiple parallel narratives on acceptable use and representation of the human body (Congolese or Belgian) beyond its medical assignation.

In 1923, the medical chief of the Belgium Congo stated after observing the abysmal failure of colonial training programmes for indigenous medical assistants, ‘One needs not be too surprised that ignorant students, *some of whom are direct descendants of anthropophagous parents*, do not have the moral qualities…needed to be a good indigenous medical assistant.’^1^ A few years later one of the earliest indigenous converts to Protestantism, a man named Nlevmo, related the story of his conversion to Christianity in the *Baptist Missionary Herald*. As a young boy Nlevmo was ‘given’ to pioneer missionary W. Holman Bentley in 1881 upon his arrival at the Stanley Pool (now Malebo Pool). Nlevmo became an important translator and assistant for Bentley, travelling with him to England and Holland before returning to Bolobo Congo to work at the British Baptist mission. When his father told him as a young boy that he would be going to live with Bentley, Nlevmo was terrified, as it was thought ‘the white men sometimes ate the Congo people’.^2^ As these two anecdotes reveal, the imagined threat of cannibalism often lingered just beneath the surface of both sides of the colonial interchange.

Luise White has intensively studied rumours of arcane creatures such as vampires and cannibals in colonial Africa, arguing that such stories reveal ‘all the messy categories and meandering epistemologies many Africans used to describe the extractions and invasions with which they lived’.^3^ In a similar vein to vampire stories, cannibal stories can tell us about the experiences and understandings of Africans under colonial exploitation. But fantastic myths were not the exclusive domain of Africans; colonial writing is rich in fables presented as fact, with one of the most common tropes being that of the savage African cannibal. Thus, such rumours cannot be reduced solely to allegories of extraction and exploitation since both coloniser and colonised propagated stories of the other’s cannibalism. Stories of cannibalism are linked to specific acts on the body, and understandings and misunderstandings linked to these acts.

This essay looks at the narratives surrounding the specific act of cutting the body in the Belgian Congo – the internal bits and pieces made visible – to reveal what Congolese people^4^ or colonisers understood of the body when its insides were exposed. This research is part of a larger project on anatomy and the body in the Belgian Congo, conceived on a wide scale. During my search for data on cutting and obtaining bodily samples, intriguingly, references to anthropophagy unexpectedly continued to surface in various types of archives: official government reports, medical school records, ethnographic studies, medical missionary correspondence, etc. In many cases, it was an exercise in alterity, but these references were neither confined to exoticising accounts nor to colonial observers. They were, however, always associated with specific practices on human bodies. Within the colonial setting, where plural societies^5^ co-existed and co-mingled in sporadic ways, the process of cutting the body in particular, whether living or dead, lent itself to conflation with cannibalism and other fantastic consumption stories by both Congolese and Belgian observers.

Historians rarely bring together ‘fantastic consumption’ stories by colonised populations and those by colonisers, although these are often linked phenomena, as this article will show. While intimately tied to issues of alterity and deontology, such tales also reveal crossed readings relating to the natural world and ontology. Further, nothing confuses the nature/culture divide and the myriad analytical constructions to understand this divide more than the human body. It is both natural and cultural, thing and representation. The human body at the point near or after death is even more intractable as an analytical subject/object. This essay will map out different views of human materiality and personhood through medical technologies of opening the body. These disparate readings of the body make visible epistemological conflicts that arise when a person cuts the flesh of another in the space between life and death.

The focus here on specific bodily practices has two main goals. The first goal is to sideline, for a moment, what ‘medicine’ is, and examine the related, but somewhat distinct, question of what the body is and what makes it ‘dis-eased’. This is, primarily, methodological experimentation with a framework that permits analytical parity in comparing medical practices by groups with grossly unequal socio-political power. In the field of colonial medicine, the ever-troubled question of the relationship between biomedicine and traditional/indigenous medicine has been treated either by showing how biomedicine was constituted from traditional medicines, how biomedicine is culture-bound (presumably like traditional medicine), or how traditional medicine is rational and objective (like biomedicine). To use the language of Science and Technology studies (STS), comparative parity is often achieved by reading traditional medicine as more ‘objectivist’ or biomedicine as more ‘constructivist’. Both are both, clearly. But they are also very different. The second goal of this paper is to map the nuances of these similarities and differences, but beyond the objectivist-constructivist dipole. Readings of the body, colonial and Congolese, are both culturally determined and empirically based. In relating these readings, the narrative also sketches new perspectives on a particular historical moment (arguably the primordial goal of historical research). Like the *histoire croisée* framework, the methodological approach of this paper attempts to avoid the weakness of the over-synchronic framework of comparative studies and the inherently diachronic nature of transfer studies by looking at the intersecting of the historicity of the object and the approach to the object.^6^

The Belgian Congo had several indigenous political chiefdoms incorporating numerous ethnic groups, with a wide range of practices and understandings of the human body, the corpse, and death. Even given this diversity, we can still delineate a broad shared understanding of death and the afterlife in central and west Africa where: the corpse was not entirely deprived of agency after recent death, the recently dead had an active role in the lives of the living, and certain body parts had power separate from the integrated whole that lingered after death.^7^ These views are not unique to the African world, as religious scholars in Western contexts are well aware. But, scientists coming in to work in Africa at the very least aspired to have a contrary view, and to proselytise this view to their African workers, patients, and converts. (This proselytisation was often a conversion in the religious sense as well, as the work of medicine in the Congo was confided to various religious orders along with trained secular medical doctors – but this is another story.)

I am focusing on the field of medicine, where attempted conversion was largely flowing in one direction, from the colonist to the colonised.^8^ We will look first at a theoretical/ideational view of anatomy before moving to concrete practices of surgery and autopsy. Western medicine primarily existed in the Belgian Congo in three forms: state medical programmes and institutions with large numbers of Catholic missionary auxiliaries, Protestant missionary medicine, and private industrial medical provisioning for company workers. Public health was the realm of state medicine; surgical interventions were common in Protestant missionary hospitals as well as state hospitals, and to a lesser extent within private concerns. Medical establishments were central to the colonial project, and medical staff constantly strove to convert the indigenous population to a specific conception of health and hygiene. *Convert* is a deliberate term, as colonial educators knew that they were not simply sharing their knowledge with the local populations but rather attempting to eliminate and replace existing ontological understandings with another way of valuing the human body and the human being. As in religious conversion, doctors and other health workers and officials could not accept that a convert to medical science could also continue to believe in witchcraft or rituals.

## 1 Anatomy

A dependence on the truism that ‘seeing is believing’ can be found in the early medical proselytisation in the Congo. Early medical officials had great faith in the self-evident visibility of the natural fact. This was expressed most clearly in a general reliance on the optical research instrument *du jour*, the microscope. One doctor wrote, ‘the native who *has seen* the causes of disease in the microscope…will be able to convince natives of the uselessness of fetishism, and put an end to the pernicious practices of sorcerers’.^9^ Indeed, there was a sort of mini microscope mania in the 1910s and 1920s in the Belgian Congo, with dozens of missionaries and many an administrator, along with the medical staff, trained in seeing diseases (particularly the sleeping sickness trypanosome) under the lens and sent into the field with their government-supplied microscope.^10^

However, the microscope would not be the path to converting indigenous peoples to modern medicine and Christianity. To ‘see’ correctly with a microscope required expertise in preparing samples and in interpreting what was seen. Although there was much talk about how the microscope would convince indigenous people through the incontrovertible evidence that was seen, different users saw different things under the microscope. Even of the European men and women trained in the microscope, few learned to see much more than the infamous sleeping sickness trypanosome during their month-long laboratory training. Further, seeing the micro-organism did not mean that the truth of microbiology became evident: ‘believing’ did not so easily come from ‘seeing’. Gross human anatomy, as an order of magnitude greater in size and unmediated by a scientific instrument, would on the surface appear to lend itself more to being seen in the correct way. Western medical culture claims to visualise the human body in a de-sacralised manner, particularly its internal workings.^11^ Anatomical figures represent the human body, but they represent the human body to a specific audience that has learned to see what it is seeing in the intended way. Much like a geographical map, these representations require a shared semiotic code to be read correctly.^12^ In other words, viewing the anatomical model as an ‘objective rendering of the body’ depends on a vast array of cultural assumptions.^13^ Nonetheless, this particular visual reading of the human body, a Western anatomical understanding, was considered crucial to effective medical training. When the Belgian colonial government began training its first generation of indigenous medical assistants, it provided top-of-the-line microscopes and anatomical models to facilitate a Western visual understanding of the human body, to little avail.

The first training school of indigenous medical assistants (*école d’infirmiers*) was created in Boma the year after the transfer of the Congo Free State from King Leopold II to Belgium in 1907. This school was equipped with microscopes, as well as a ‘natural’ life-sized mounted skeleton, a collection of 13 anatomical plates mounted on wood, and a headless paper maché trunk with removable lung, heart and organs, showing the stomach, liver, spleen and intestines. In 1920–21, under the urging of the Minister of Colonies, the system was organised and expanded to five schools (with Boma being transferred to Leopoldville): Leopoldville, Coquilhatville, Stanleyville, Ibembo and Elisabethville. The training programme lasted three years, with the first two years focusing on deontology,^14^ hospital service, parasitology and hygiene. Human anatomy was taught in the second and third year.^15^ Even in this early period, one instructor noted that ‘an education entering into details of physiology and human anatomy…will not have the slightest chance of being understood by Negros [*noirs*] who are already frustrated, many of whom can barely read or write’.^16^

A basic medical instruction book was commissioned and written by a Dr David, and translated into Kiswahili in 1925. This handbook was carefully designed for the Congolese *infirmier*,^17^ with 250 pages of text and a blank page between each for note-taking. The introduction informs the student that to become a doctor in Europe required many years of learning, and ‘[t]he *infirmier* can’t learn all of this; he isn’t a doctor, he *helps* the doctor, he does what the doctor tells him to do’. David belabours the fact that the *infirmier* must have many exceptional qualities and he ‘should understand that he was chosen to be a student because he is more intelligent than his fellow natives and will use his knowledge to help them’.^18^ The book then passes on to a lesson in anatomy, replete with mechanical analogies. The textbook introduces the nervous system by explaining that ‘our body is like a machine, but our machine doesn’t need anyone to turn it on or direct it. It is the most perfect part of our body that commands the other parts. This superior part is called the *nervous system*’.^19^ Or, for digestion, ‘to run, a machine needs fuel, coal. Our body needs various types of fuel’.^20^ As for respiration, ‘in order for a machine to run, it is not enough to fill it with wood; the wood won’t burn if no air is around. This is the same in our body; air is as necessary for life as food is’.^21^ Whether intentional or not, the textbook presents the body in a similar way to contemporary hygiene textbooks for children in the Belgian metropole. Further, it presents the body-as-machine metaphor as evident. But this metaphor, central to the mechanical philosophy that predominated the modern era in Europe, is highly contingent on a particular moment in European history. While considerable literature exists on the rise of mechanical philosophy^22^ in the European context and the subsequent decline of other ways of understanding nature and human biology in the early modern era, the particularity of such views can be overlooked when historians analyse the spread of Western science in the imperial domain, as if the dynamics of colonialism overshadow the contested nature of epistemological understanding. But, the Western world did not become ‘disenchanted’ because mechanical philosophy was the most effective or true way of explaining the natural order of things; disenchantment in the colonial world was another thing altogether. In the end, David’s book, alienating in both tone and presentation, was the only commissioned handbook for indigenous medical training by the state in the early colonial period.^23^

The colonial government ordered anatomical plates for each of the five new *infirmier* training schools, at a considerable price (12 500 francs). These pieces included, for each school, a full-sized human *écorché* (anatomical model with skin removed to display musculature) with removable organs and an opening heart, a varnished plate on canvas of insects that were vectors of tropical diseases and four additional anatomical plates of the human digestive system, the circulatory system, the respiratory system and the nervous system. Students were also to assist with autopsies whenever possible, ‘to show them pathological legions that can explain certain morbid symptoms for them. [T]hese autopsies will also serve as an occasion for an anatomy lesson’.^24^ The anatomical section of David’s handbook served as a narrative companion to the anatomical plates.

Initially, the push for medical training and the presumed accessibility of scientific knowledge came more from administrators than medical personnel, but colonial medical staff were enthusiastic collaborators in this early stage. This quickly changed, when the medical staff found their role as both supervisors and teachers to the Congolese workers more difficult than anticipated. A standard litany of complaints arose in the medical reports of lazy, illiterate and dishonest students. Very few students could pass the exams of the first year, much less arrive at obtaining a certificate.^25^ Doctors grumbled that the ‘feeders’ into this system, the missionary fathers, gave them the worst students and sent the better ones into clerical service.^26^ In the second year, 10 of 13 students failed. The instructor noted that this was despite a well-equipped classroom, with an homme ecorché, a skeleton, several anatomical plates and four microscopes. He grumbled further that Dr David’s instruction booklet was ‘still for most of them an incomprehensible gibberish that they are unable to understand or translate into their own language’.^27^ Within three years, the Governor General sent a deeply pessimistic report on these schools to Brussels, prompting the Minister of Colonies to rebuff this negativity. He emphasised the absolute need for the colonial service to continue to improve their functioning.^28^ Belgium had neither enough men nor money to staff the entire Congo medical service with expatriates. Still, two of the schools were shut in the first few years. After a decade, results were so dismal that another re-organisation was attempted, with no improvement in outcome.

The medical chief observed after proctoring the end of year exam in 1923:

[I] …was disappointed to see how many students memorised by heart without understanding. They recite these litanies that mean nothing to them. The anatomical murals, however suitable, *represent nothing to their objective brains* [*cerveaux objectifs*]. Dr Van Diest of Buta, struck by this fact, began teaching them anatomy with freshly killed animals. In terms of splanchnology, I believe this is the best way to proceed.^29^

It is interesting to note that the possibility of human dissection for pedagogical purposes is not considered. This may be because autopsy was already available as a tool of training, or health regulations were in place that mirrored those in the metropole favouring autopsies over dissections.^30^ But it also may be due to the political climate and desire to avoid further speculation on exploitation of the black corpse, the tropical climate itself and quick spoilage of any corpse, or the belief that such a resource would ultimately be wasted on students of such low capabilities.

The medical chief’s comment gives us a glimpse of what the medical personnel did not see directly, namely the vast epistemological disconnect in anatomical knowledge. For him and others like him, the ‘objective brains’ of the indigenous people were the problem.^31^ They had not learned to take conceptual leaps and could not conceive of an anatomical model as analogous to the human body. Because the ethnic make-up was quite mixed in Stanleyville, we cannot be certain to which ethnic groups these students belong. However, as observed in by Steve Feierman, medical systems in sub-Saharan Africa do not map neatly onto ethnic identities.^32^ We have a study of indigenous healers (*nganga*) of the Kongo ethnic groups from which we can extrapolate some parallels. While this study was undertaken in the 1960s, we can assume that there is considerable conservation of some theoretical notions from twenty years earlier. This study describes Kongo anatomical knowledge as ‘a symbolism that references across domains (natural, social, cosmological) that amplify and explain experience’.^33^ Further:

It is difficult, if not impossible, to abstract a simple physical ‘anatomy’ from the *nganga’s* thinking, because… organs, functions, and bodily symptoms are related to a more expansive unit than is the case in Occidental medicine, philosophy, or religion… [*Nganga*] ideas about the body… actually embrace constant reference to social relations, plants, and medicines. As has been suggested, the heart stands in analogous relationship to organs and functions, foods, medicines, human beings, spirits, and the whole universe, like a signal – calm in health, agitated in illness – for the *nganga*.^34^

As an example, the author describes the concept of the abdomen:

The *vumu*, abdomen,… is analogous to the lineage house in society, just as the heart is analogous to the chief. *Vumu* thus embraces both physical and social referents. It denotes vulva, uterus, pregnancy, mother’s breast, lactation, and abdomen, as well as family, or descents of the same mother… it is a verbal category of congruent ideas, both physical and social, drawn from a dominant idea about subsistence, identity, and well-being.^35^

This is a referential and reverential anatomy that does not easily align with the anatomical plate. Roland Barthes observed that ‘no sooner is a form seen than it *must* resemble something: humanity seems doomed to analogy’.^36^ But the analogy is structured by the context. The anatomical plate is not a self-evident fact. If the health of the body is never entirely reducible to the material, and vital qualities of the living linger in the ’newly dead’, how does a paper mâché figure represent a human body? If disease is linked to the relations of power within the body itself and not the material substance, studying the plastic, paper mâché, or wooden object serves little purpose. Further, the anatomical plates artificially separate different functional systems (digestive, circulatory, etc.), excluding parts not central to that particular system. If indigenous anatomy was more expansive than its biological counterpart, Western anatomical models were radically more reductive. Linking the two through the freshly killed animal was perhaps the best compromise in reaching an understanding between two systems of representation in a context where human dissection for instructional purposes was not practicable.

These epistemological issues are also complicated by language, and questions of interpretation and translation. There were dozens of major and minor languages in the Belgian Congo. While the medical textbook for students was translated into Kiswahili, this language would have been as incomprehensible as French or Dutch for many students. Even between two languages, a translation of a simple term could be extremely complicated. As an example, in a British Baptist missionary station in Upoto, a nurse recounted in the mission newsletter the history of a village woman coming into the dispensary complaining of pain. This village woman was also one of the students in the new hygiene and physiology courses offered by the mission. When the nurse asked her where the pain was, she pointed vaguely to her body and said ‘there in my heart’. The nurse replied, ‘But haven’t I been telling you this morning that your heart is not there but your *likundu’.* *Likundu*, the only word in the nurse’s letter written in the vernacular Bolobo, is parenthetically defined as the ‘stomach’. When the nurse told her she had a *likundu*, the villager panicked and ‘gave a wild look of terror round the waiting room and gasped, ‘Mama, you surely don’t say that I have a stomach’. The nurse went on to explain, ‘The native belief is that only witches have stomachs and in the old days the unfortunate victim convicted of witchcraft was opened up to prove the statement’.^37^

Here, we must question how the villagers could misinterpret the hygiene training to conflate the Bolobo word (*likundu*) for stomach with the word for witchcraft substance (to be discussed shortly), which was, according to one scholar, *likundu libe*.^38^ However, a contemporary Protestant missionary who published extensive ethnographic studies on the Boloki and Ba-Kongo ethnic groups translated *likundu* as an adjective for witchcraft or craftiness, with attributes such as ‘*nganga ya likundu*’ (witchcraft doctor), *likundu* charm (cleverness charm), or ‘*awi na likundu*’ (to die by ones’ own witchcraft).^39^ In the Bateke region of the Belgian Congo, some 100 kilometres to the west, the Tio word for stomach and witchcraft substance were sometimes both referred to as *ifi* and sometimes as *ifi* and *impfiri* respectively.^40^ Perhaps a similar sort of conflation occurred in Bolobo. Thus, the villager’s confusion could be either an issue of translation or interpretation, depending on how the stomach was explained (both geographically and functionally) to the class and how the conversation in the dispensary was actually conducted. Did the villager misunderstand the medical lesson, or did the nurse misunderstand the villager? It is also important to note that the nurse failed to explain that the ‘opening up’ of the suspected witch was done after death, as we will see. The narration confuses and omits to make this particular exchange more exotic for the imperial reader, but it is also likely that confusion and omission was unavoidable in the complicated reconfigurations of the human body occurring in the Belgian Congo.

## 2 Surgery

With some absurdity, a Catholic Father would observe of his Congolese charges in 1891, ‘Anatomy, as they say, has largely fallen into disuse since the arrival of the white man bringing civilisation…. From the moment when one could no longer eat people, how could one learn to dissect them?’^41^ While such a statement was clearly spurious, cutting into human flesh was known before the arrival of European surgeons, but its extent and purposes were radically different. African cutting rituals existed for purification, as well as for medical treatment involving small incisions on patients with ritual removal of offending objects (which could range from stones or dried spiders to live worms).^42^ Scarification and tattooage, or body inscriptions, fall into another category of cutting, because of their non-therapeutic ascription and their superficiality (they do not break the surface of the skin), and are excluded here.^43^ While African surgery to repair physical injury was known, it seemed to be rarely used.^44^

What was biomedical surgery to the Congolese? It was certainly new, and it was fascinating. Early on, it was a public spectacle, whether the surgeon desired it or not. In some cases, surgery was staged as a public performance to awe the local populations.^45^ In other cases, it was public to make the actions of the surgeons transparent, and thus dispel any nefarious rumours of cannibalism or witchcraft.^46^ In most cases however, early surgery was public only because privacy could not be enforced. A Protestant missionary doctor describes to his American audience the ‘syringe-boy’, a uniquely Congolese staff member of his surgical team in Banza Manteke in 1919:

A visitor to my clinic seemed to be interested in a student doctor that seemed to have little else to do during the operation than holding a glass piston syringe in his hand, the syringe being filled with water. My friend soon had his curiosity rewarded for all at once, quickly and silently, the student stepped to the wall and inserting the business end of the instrument into one of the many spaces between the clap-boards that afford fine opportunities to curious outsiders to see what is going on in-side, pushed the piston home, this water being forcibly expelled into the eye of the peeper…we could not enjoy any sort of privacy were it not for the syringe-boy.^47^

Even as late as 1936, in the newly constructed hospital at Pimu, a British Baptist missionary doctor described his first surgery of a man gored through the abdomen after an elephant hunt gone wrong:

They have brought him to hospital and I operated but I felt he was past any aid we could give him except that of the control of his pain with morphia. He died three hours later. That was my surgical debut here. It was a dramatic operation done in a very primitive and elementary style with 60 or 70 spectators all standing round in dead silence, watching.^48^

We need not question too much why surgery would draw spectators. Seeing the inside of a human being, and a living human being at that, was an exceptional event, and exceptional events attract interest. A more complicated question is how patients approached undergoing surgery.

As minor interventions often radically improved medical conditions that seemed hopeless, surgery’s popularity grew rapidly in the colonial period, even if it was also suspected of having a cannibalistic aspect.^49^ But, some of this demand was linked quite closely to the conditions of the colonial state, as in the case of hernia surgery. The distribution of types of surgeries varied between the Protestant missionary and the state operating theatres, but hernias represented from half to the majority of major operations by the state in most years, and in some cases representing over 80% of all major operations.^50^ While hernias may have had a high prevalence in some populations because it could exist as a chronic condition, it seems likely that the strenuous demands of colonial labour contributed to this widespread health problem in the Belgian Congo. In the region of Pawa (Province Orientale), in 1934, an initiative to screen and treat hernias in the area unearthed thousands of cases, deriving from the demands of work in the cotton fields and cotton transport under the colonial plantation system.^51^ The sole surgeon of the Red Cross decried the screening initiative, as he could not come close to meeting the demand for reparative surgery, and almost all diagnosed cases were left untreated.^52^

Whether all surgeries were entirely voluntary is unclear, but colonial medical officials commented repeatedly, and in different contexts of the Congo, that once the proof of surgery’s efficacy was seen, its popularity grew rapidly. This was also affirmed further with missionary hospital correspondence, where intermittently visiting surgeons had waiting lists for patients that reached six or eight months.^53^ One doctor even called surgery ‘bait’ [*appât*] to draw recalcitrant indigenous people to Western medicine.^54^ Ultimately, more surgery was desired than could actually be provided. It was possible that the technical spectacle of surgery inspired awe, and surgical interventions could be seen as analogous to African rituals: purificatory as in some African cutting rituals, or proof of innocence as in survival in a witchcraft poisoning ordeal.^55^ However, we cannot lose sight of the fact that the Congolese also assessed empirical evidence. Certainly surgery was not popular everywhere it was introduced in colonial Africa.^56^ Undoubtedly, surgery in the Congo was in demand (and awe-inspiring) not merely because of its theatricality but also because of its results. As one missionary wrote, ‘the wonders wrought by surgery are truly a marvel to these people. In July last, … a doctor performed a slight operation upon a boy who had shot himself in the eye. The people around looked in wonderment upon it, and called him ‘Great Doctor’ and crowded to see him.’^57^ As another doctor noted, ‘Surgery is perhaps the most spectacular department of medical works…If surgery effects a cure, the result is very apparent. When someone who previously had a large and obvious lump returns to his village without it, the power of European medicine cannot be gainsaid’.^58^

Surgery in both the Congolese and Belgian context was done to prolong life. If there was extraction of physical objects from the body, it was *not* metaphorical in either context. An actual object, be it bullet, tumour, appendix, worm, bone or charcoal, was withdrawn with the – ideally – concomitant reduction in the suffering of the patient. In the Congolese context, it may have been ‘trickery’ on the part of the practitioner, but the patient may not have viewed it as such. There is no mention of ostracisation, adulation or indeed any sort of special status attributed to post-operative patients beyond the status of a newly healed patient. What is clear is that Western surgery was seen as offering a possibility for the treatment of ailments that were previously not effectively treated by indigenous medicine. A belief in the healing power of surgery did not mean abandonment of faith in the healing power of ritual, as the resort to indigenous medicine continued unabated among those who sought surgery. Western surgery could be understood in ways aligned to indigenous knowledge of the human body and disease. New representations of the body syncretised with rather than replaced existing ones.

## 3 Autopsy

Cutting into the living was done to prolong life (biopsies were arguably an exception to this) both for Congolese people and for Belgians; cutting into the dead was another story. The dead were cut open for a variety of reasons. Most notably the profanation of the corpse could be used for post-mortem punishment of the deceased or as a means of warning the living of their potential fate in following the path of the deceased. Until very recently, the dissection or autopsy of criminals in Europe served in part this same purpose.^59^ Bodies were sometimes mutilated to obtain trophies, a common practice during many colonial military campaigns in early twentieth-century Africa, often justified on the grounds of scientific collecting.^60^ During the wars of conquest near Lake Tumba in the Belgian Congo, thousands of corpses were systematically mutilated for the very practical purpose of assuring that ammunition cartridges were not being wasted, as baskets of severed (and smoked) right hands of fallen Congolese people were presented to commanding European officers as proof of efficacy.^61^ Although these sorts of cuttings no doubt influenced local interpretations of other sorts of manipulations of the dead, here we focus on cutting the corpse to obtain information. In the West, the cutting of the corpse to retrieve information from it as an epistemic object falls under the purview of post-mortem examination in the form of autopsy or dissection. But the cutting of the recently deceased to reveal or inform is also known in the colonial Congolese context. This is best described in the well-known ethnology of Evans-Pritchard on the Azande, an ethnic group living on the frontiers of Uganda, Sudan and the Belgian Congo in the 1930s. Evans-Pritchard describes a procedure he calls ‘autopsy’ performed on the corpses of suspected witches to find ‘witchcraft substance’. This autopsy was performed in public at the edge of the grave:

Two lateral gashes are made in the belly and one end of the intestines is placed in a cleft branch and they are wound round it. After the other end has been severed from the body another man takes it and unwinds the intestines as he walks away from the man holding the cleft branch. The old men walk alongside the entrails as they are stretched in the air and examine them for witchcraft-substance.^62^

This witchcraft substance was described as lumpy black material found in the intestines. Elderly men, experienced in identifying such substance, were present to adjudicate findings. When the autopsy was completed, the entrails were replaced in the body, and the performer of the autopsy was ritually cleansed.^63^ Although the exact process likely varied between ethnic groups and regions, the examination of the corpse for proof of witchcraft was commonplace through the Congo during this time.^64^

Witchcraft substance, or another part of the witch’s body, could be powerful in itself. Thus, the living could obtain this substance and use it to harm others. More generally, the recently dead, whether witches or not, in their liminal not-truly-dead state could still have power through the materiality of their own corpse. As one observer wrote in 1930:

Someone was sick, the witch doctor was called in and after a time he said the man who had recently died was the cause; he wanted a companion. So the body was taken out of the grave, burnt & the bones thrown on the plain where some of my boys found them. Now that the body has been destroyed the spirit cannot play any more tricks!^65^

Florence Bernault argues that there is a divide between Western European and Equatorial African perceptions of the relationship of the body and power. Western Europeans perceive a metaphoric relationship between the two. In the African context,

people perceived a direct, unmediated relationship between the body and authority, as the former represented the privileged location of procedures and institutions that crafted, channeled, and controlled power. In doing so, the body was not seen as a physical reality whose existence derived from biological integrity, but as a multiple and fragmentable entity that retained power beyond death and dismemberment.^66^

Bernault further argues that this distinction is exaggerated, as Westerners actually still fetishise the body in various ways after death. One of the ways that this sort of fetishisation can be seen is through the treatment of the ‘resting place’ of the dead. In the West, the tombstone marks the resting place of the body even when it has decomposed to dust. As such, the tombstone is ‘an index’, as one scholar called it, as opposed to a sign, in that the material presence of the body gives meaning to the grave marker.^67^ In Central Africa, the grave was often not marked in a notable way, even if it was tended to and attended with some frequency, as the doorway between the visible and invisible worlds.^68^ While the Western tomb served to prolong the social role of the (recently) dead, the African grave was often a less obvious feature of the landscape, serving in a way that was much more discursive and symbolic yet still incorporating the dead in community life in various ways.^69^

Cutting of the recently dead must be read with a consideration of this strong distinction in Central Africa between what Mahaniah calls the *morts-vivants* and the *vivants-morts*, or what we can perhaps call as the recently dead and the ancestors.^70^ How were autopsies on these ‘not-quite-dead’ corpses performed and perceived in the Belgian Congo? Vansina notes that the recently dead are greatly respected, much like elders, but not particularly subject to awe or piety because they are conceived as commonplace, active participants in the world of the living.^71^ It is unclear if this would make autopsy more, or less, of a violation. It is clear, though, that the Congolese only cut the dead who were already morally suspect in some fashion – whether witch or enemy or murderer. Innocents were not autopsied.

In contrast, general autopsy was a common practice in the Belgian Congo, for both medical and juridical purposes. The larger hospitals were all equipped with an autopsy room, and assisting at autopsies was one of the reasons that *infirmier* training was unpopular, along with the necessity of touching and moving the dead.^72^ We find no similar objections to surgeries (i.e. cutting into the living) by indigenous assistants. Autopsies on Europeans are occasionally mentioned after a suspicious or puzzling death. Protestant missionary doctors were also required to perform autopsies for the colonial state.^73^ Autopsies on blacks, as opposed to whites, were often for more general research purposes on tropical diseases, as ‘harvested materials …permit necessary comparative research and the reconstruction of certain anatomical pathologies’.^74^ From 1915 to 1921 in Elisabethville, 421 autopsies were performed for the purpose of establishing a local nosological profile. The doctor noted with some pride that up to 1918 almost every deceased patient at Lubumbashi hospital (of the Union Minière du Haut-Katanga) was systematically autopsied. Also of note was that most of the deceased were men between the ages of 20 and 35 who had succumbed to pneumonia or other normally non-lethal diseases for this age group.^75^ In the laboratory of Stanleyville, 65 indigenous autopsies were performed in six months of 1929, an average number, and again most were performed for general research purposes.^76^ Like surgeries, autopsies could be forcible public performances. As one doctor wrote of the deceased, ‘without a morgue, bodies remain in the room where they died until the time they are transported… and even if an autopsy is required by the law, I am obliged to perform it in the open’.^77^ One can only imagine the effect on living patients in the same room, and the sorts of apocrypha that would arise from such an event.

The European decried the fetishism of the African, where pieces of the corpse could be kept in sacred objects made powerful by this fragment of human material. But a parallel practice existed among the Belgians, the very specific practice of cutting and taking bits of the living and dead and sending them away. With the panic over the spread of sleeping sickness in Central Africa, tens of thousands of Congolese people submitted to lumbar punctures, had their fluid removed and sent off to be studied under the microscope.^78^ Some suspected that this palpating and puncturing was in fact a way of putting sleeping sickness into their bodies.^79^ Many others had bits and pieces cut off or extracted and mailed to the School of Tropical Medicine in Antwerp, and then often sent further on to research institutes in Europe or America.^80^ Such extractions, common throughout colonial Africa, led to an entire genre of stories of blood-sucking and cannibalistic white men.^81^ In any single year, hundreds of pieces of ‘human material’ were examined in the colonial laboratories. As an example, in six months of 1929, the laboratory of Stanleyville examined 309 pieces of ‘human material’ sent from 273 necropsies at the black hospital and 26 biopsies in the field.^82^ Serious epidemics could require mass forced autopsies, as was the case with a plague epidemic near Lake Albert in this same year. During this epidemic, autopsies were performed on suspected dead until the supply of gloves ran out, with several bodies exhumed and autopsied after ‘unauthorised’ burial.^83^

Administrators exasperatedly recorded ‘irrational’ rumours circulating when they explained to their superiors the recalcitrance of indigenous populations towards colonial medicine, never allowing that these rumours could have any basis in reality. However, rumour could be made true. In a case reported in the early post-colonial period ‘One nurse’s aide and pathology assistant in a mission hospital… sold small pieces of cadaver as a power fetish, reinforcing the fear that in performing autopsies (i.e., tampering with the dead), the missionaries ‘ate’ parts of the body to strengthen themselves’.^84^ Here was a rumour that was true, with acts partially driven by indigenous beliefs, which furthered a particular narrative of foreign, medical consumption of the body.

Autopsies were not done for the good of its subjects (who are dead and thus presumably according to Western science beyond aid), but for medical or juridical research. The material body was being opened up to further knowledge. But that knowledge itself was also the basis of a certain type of power (to paraphrase Francis Bacon) and authority: scientific authority. The recently dead human body, while it maintains its ‘thingness’, is useful because of its similitude to the living human body. A badly decomposed body provided little useful information on what it was or what caused its death, and bones were not autopsied (although they were collected). Among the Congolese, a similar logic was at work, as the corpse remained a powerful social actor while it maintained its human appearance. The more life-like it was, the more it could act in the ‘visible world’. Destroying the body mitigated the means by which the recently dead could act in the world. The category of ‘recently dead’ was not stable for either the Belgians or the Congolese. The colonial government ‘knew’ that indigenous tampering with the human body was not for scientific purposes, and thus labelled it profanation of the corpse and declared it illegal. Indigenous populations ‘knew’ that what the colonial doctors did was also profanation of the corpse, as the new corpse was still human, still a part of culture, still sacred. Colonial doctors would have protested that the dead body *as they used it* could not be profaned. It is a ‘thing’ alone, and thus already ‘profane’. They rejected the perception of the ‘not-quite-dead’ body as preposterous. Still, Congolese manipulations of the dead body, or pieces thereof, were immoral violations of the dead. In this reading, the dead become sacred again. Through the double standards of colonialism and Western science, the acceptable, moral treatment of the corpse became contingent on both Christian morality and biomedical exigency.

## 4 Consumption stories

In Central Africa, it is common knowledge that a witch from a distance ‘eats’ the soul of the various organs of his or her victim’s body, these organs die, and eventually the body dies. It is not metaphorical, even though it is not tangible.^85^ This act cannot be seen; only its secondary effects can be viewed in the illness of the individual being consumed from the inside. Again, it is literally cannibalism, but not a physical cannibalism. But to understand it as such is to understand that the body is something more than material. European stories *of indigenous stories* of cannibalism fail to realise this. Did the eating of the physical body exist? Most certainly to some extent, but I suspect many of the stories of cannibalism circulating in colonial circles in the early twentieth century were of the non-physical variety, misinterpreted by colonial observers. Did the European ‘eat’ pieces of the colonised? No. But they did cut off, extract and send away blood, lumbar fluid, tumours and so forth, consuming them in the process of medical research. This was done on the dying and the dead, thus the correlation between such extraction and death was a close one for the Congolese. Further, the related European project of collecting bones, and ethnographic fascination with cannibalism itself fuelled such rumours. These rumours then fuelled competing myths of the others’ cannibalism. For example, in the late nineteenth century, avid skull collector and Congo explorer Georg Schweinfurth found skulls so easily attainable in the Congo that, for him, this was ample proof of African cannibalism. At the same time, he spent considerable effort attempting to explain phrenology to the very populations he collected from, to allay the growing rumours that he himself was a cannibal.^86^ The example of scientists creating their truth could be seen even more clearly in the case of Scottish naturalist James S. Jameson. This enthusiastic naturalist bought a slave child and paid Zappo Zapp’s men to kill and eat her (at a total cost of ten handkerchiefs) as part of a ‘scientific’ fact-collecting process.^87^ European cannibal-seekers both created the cannibals they sought and became cannibals themselves. Cannibal tales can be about humans literally eating humans, but they are also stories of humans consuming each other in many other ways.^88^

There was a deep disconnect between epistemological understandings of the body, ontologies of the human self, and subsequent interpretations of the practices surrounding the body, as it moved from living to dead in the Belgian Congo. In a context where social and medical death are defined so differently, terms such as ‘desecration’, ‘biopsy’, ‘fetishism’, ‘cannibalism’ and so on are meaningless without careful contextualisation. Technologies of cutting the flesh were interpreted in fantastic and nefarious ways by both coloniser and colonised. This is not an issue of compare/contrast, but rather a problem of multiple parallel narratives on acceptable use and representation of the human body. These multiple narratives allow us to understand the value of such techniques (Congolese or Belgian) beyond their medical assignation.

The human body is powerful. To label this power as symbolic or objective is, sometimes, an irrelevant distinction, as this power has concrete effects on social relations. Both intruders and indigenous people used the tools that they had available to either syncretise or delegitimise alternative readings of the body. For the Congolese, rumour was key. Rumour, as James Scott and others have shown, is one of the classic ‘weapons of the weak’ to fight against hegemonic impositions by the powerful.^89^ Rumour-mongering may not be conceived as a political act by those who relayed these stories in the Congo, and it is not deliberately undertaken to undermine the official narrative. But rumours provide counter-narratives that are often more satisfying explanations of unusual events than the official narrative. Congolese people accepted some acts on the body, such as some surgeries or biopsies, without having to accept alternative meta-narratives around the act. It was from the acts that they could not accept but could not prevent, such as autopsies, that the greatest rumours arose. In the Belgian context though, the consumption stories that proliferated about the Congolese were often more deliberate. These stories justified the colonisation, dehumanisation and exploitation of a people who were narrated as inferior, in a variety of ways. In contrast to the Congolese, the Belgians also had legal, political, military and economic techniques to enforce their readings. Despite this arsenal, the majority of the population did not abandon their understanding of disease and its relation to the human body. Biomedicine and witchcraft continue their co-existence to this day in West Africa, because both continue to serve important objective and symbolic functions in society. Acts on the human body – visualising, repairing or dismembering – are always purposeful. An examination of these practices allows a clearer vision of how the body is read as a social or a biological text.

It is not argued here that biomedicine and witchcraft are equally efficacious or even equally robust as rational explanations for human functioning. But they both have their mythologies, assumptions and failings. Catherine Mabie, a missionary doctor who spent over forty years in the Congo, told a story of her arrival in Banze Manteke in 1898. She performed a successful surgery on a minor chief who had been wounded by a wild buffalo. He was wearing a fetish on his arm that protected him from attack by wild animals, and she was interested in collecting it as an ethnographical piece. Not wanting him to think she believed in it, she asked him what it did, and observed that apparently, it did not work and was thus worthless. Would it not be better to discard it? To which the chief responded, ‘No…they don’t always work. But neither does your medicine. A man died here in the hospital yesterday’.^90^

### 4.1 Archives

AA –Archives Africaines, Ministry of Foreign Affairs, Brussels

ABHS – American Baptist Historical Society, Atlanta, Georgia

BMS – Baptist Missionary Society, Oxford

ITM – Institute of Tropical Medicine, Antwerp

RMCA – Royal Museum of Central Africa, Tervuren

## Footnotes

1.
Comments attached to the 1922 Annual Public Hygiene Report for the Belgian Congo, found in the African Archives of the Belgian Ministry of Foreign Affairs (hereafter ‘AA’), collection on Hygiene, file 4555 ter (noted hereafter as AA Hygiene 4555 ter). Folder for years 1923–26. My emphasis. The African Archives are currently (2016) in the process of being transferred from the Ministry of Foreign Affairs to the Belgian Central State Archives, thus file numbers and descriptors may change.

2.
Baptist Missionary Society Archives (BMS), Oxford England, file A/124, station reports of missions in Belgian Congo. This quote is found in the February 1926 issue of *Baptist Missionary Herald*, a monthly mission newsletter to supporters in Britain.

3.
Luise White, *Speaking with Vampires: Rumor and History in Colonial Africa* (Berkeley, CA: University of California Press, 2000), 5.

4.
There was no ‘Congolese’ before colonialism. There is a Kongo (or Ki-Kongo) ethnic group that was distributed across Portuguese, French, and Belgian colonial Africa near the mouth of the Congo River. I refer here to the indigenous populations living within the boundaries of what was the Congo Free State (until 1907), later the Belgian Congo (1908–60).

5.
This is a concept originally formulated by John Furnivall for colonialism in South East Asia, particularly for British colonialism, where different societies only interacted meaningfully in the marketplace. John Sydenham Furnivall, *Colonial Policy and Practice: A Comparative Study of Burma and Netherlands India* (New York: New York University Press, 1956).

6.
Michael Werner and Bénédicte Zimmermann, ‘Beyond Comparison: Histoire Croisée and the Challenge of Reflexivity’, *History and Theory*, 45, 1 (2006), 30–50. The *histoire croisée*approach has its strength in comparing intellectual traditions and practices during exchanges between national cultures, particularly when the researcher/historian is embedded within one culture. While its strength is in associating ‘social, cultural, and political formations, generally at the national level, that are assumed to bear a relation to one another’ (p. 31), it does not address such exchanges in environments where such formations perhaps do not bear much relation to each other, and the power dynamics are so unequal that they strongly shape outcomes of such exchanges. I am nonetheless grateful to an anonymous reviewer for providing me with this reference, as this approach provides much food for thought.

7.
Steven Feierman and John M. Janzen, *The Social Basis of Health and Healing in Africa* (Berkeley, CA: University of California Press, 1992); Kimpianga Mahaniah, *La Maladie et la guérison en milieu Kongo: Essai sur Kimfumu, Kinganga, Kingunza et Kitobe* (Kinshasa: EDICVA, Département de la recherche, Centre de vulgarisation agricole, 1982); Kimpianga Mahaniah, *La Mort dans la pensée Kongo* (Kinshasa: EDICVA, Département de la recherche, Centre de Vulgarisation Agricole, 1980); Kimpianga Mahaniah, ‘The Religious Therapeutic Functions of the Cemetery for the Kongo of Zaire’, *Religions Africaines et Christianisme*, 11, 23–4 (1978), 107–24; Florence Bernault, ‘Body, Power and Sacrifice in Equatorial Africa’, *Journal of African History*, 47, 2 (2006), 207–39; Florence Bernault, ‘Carnal Technologies and the Double Life of the Body in Gabon’, *Critical African Studies*, 5, 3 (2013), 175–94; Megan Vaughan, *Curing Their Ills: Colonial Power and African Illness* (Stanford, CA: Stanford University Press, 1991); John M. Janzen, *The Quest for Therapy in Lower Zaire* (Berkeley, CA: University of California Press, 1978); Jan Vansina, *The Tio Kingdom of the Middle Congo, 1880–1892* (Oxford: Oxford University Press/International African Institute, 1973).

8.
There was the occasional colonial interest in indigenous pharmacopeia and certain indigenous remedies, but this was more an issue of extraction than exchange. In other words, it was not so much an effort by indigenous experts to expand their knowledge as an effort by colonial administrators to obtain knowledge they considered useful. See, for example, Ern. Viaene and Fern. Bernard, *L’Art de guérir chez les peuplades congolaises, extrait du Bulletin de la Société Royale Belge de Géographie* (Brussels: Berqueman, 1911).

9.
AA Hygiene 4555 ter, folder for 1920–1923. Note, unsigned, comparing various indigenous medical training schools in other colonies to those in the Congo. This folder contains discussions of indigenous medical education in the colony. Emphasis in original.

10.
A review of the correspondence of the directors of the École de Médecine Tropicale (at the Institute of Tropical Medicine Archives in Antwerp) and the hygiene files of the Archives Africaines turns up numerous requests for microscopes, as well as correspondence on microscopy training and on the purchase of microscopes for missions as a condition for laboratory training of missionaries. The Royal Museum for Central Africa (RMCA), Tervuren, photo archives of the Belgian Congo also contains dozens of photos of men and women peering into the microscope.

11.
I say ‘claims’ as the literature amply demonstrates that human remains used in anatomical preparations continue to be subjects ‘post-mortem’, their objectification never truly achieved. Myriam Nafte, ‘Institutional Bodies: Spatial Agency and the Dead’, *History and Anthropology*, 26, 2 (2015), 206–33; Margaret Schwartz, ‘An Iconography of the Flesh: How Corpses Mean As Matter’, *Communication*  *1*, 2, 1 (2013), 1.

12.
Maps and semiotics are studied extensively enough together to have their own discipline, ‘cartosemiotics’.

13.
See the essays in: Martin Kemp and Marina Wallace (eds), *Spectacular Bodies: The Art and Science of the Human Body from Leonardo to Now* (London: Hayward Gallery, 2000); Schwartz, *op. cit.* (note 11), 1.

14.
Deontology, or the original French *déontologie*, denoted professional manners or professional code of conduct.

15.
AA Hygiene 4555 ter, 1907–1913 file with correspondence between the Health Services, the Minister of Colonies, the Secretary of State, the Governor General and Robert Drosten, a specialist in medical instruments living in Brussels charged with ordering medical supplies for this new school.

16.
AA Hygiene 4555 ter. Unsigned note, likely written by the colonial health services director (*médecin en chef)*attached to a letter dated 19 August 1920 on the organisation of these training schools from the Governor General of the Belgian Congo to the Minister of Colonies.

17.
I retain the French word because of its specific connotation in the colony of a male medical assistant that is some combination of nurse, orderly, and laboratory assistant.

18.
Dr David, *Vade-Mecum à l’usage des infirmiers & des assistants médicaux indigènes* (Brussels: Vromant, Royaume de Belgique – Ministère des Colonies, 1922), 7–8. Emphasis in the original.

19.

*Ibid*., 21.

20.

*Ibid*., 29.

21.

*Ibid*., 39.

22.
In brief, nature could be described by mechanical laws, analogous to a perfectly working clock created by a distant Almighty Clockmaker. The human body also worked like a machine, even if, as argued by Descartes and others, still directed by the immaterial mind.

23.
The American Baptist Missionary Society also began a school for medical assistants along colonial guidelines in Sona Bata, Belgian Congo. The school began in rudimentary form in 1932, its diploma recognised by the Belgian Colonial State in 1935, with the first official class graduating in 1936. Catherine Mabie, an American Baptist missionary, had written a small medical manual for assistants in 1927, which may have been used in this school. More likely, CC Chesterman’s *African Dispensary Handbook* (1929) was used, or something like it. Chesterman was a doctor with the British Baptist Missionary Society. Unlike David’s vade-mecum, Chesterman’s book does not cover anatomy, as he assumes that students using his book already have the necessary basic understanding of the human body. Unlike the tone of David’s explications, instructions in the African Dispensary Handbook are terse and step-by-step, with no analogies. The book is illustrated with beautiful colour plates of microscope images, as well as insects. The only human bodies represented are a few graphic photos of living patients the end of the book illustrating common ailments. The only images in the section on surgery are those of surgical tools. Clement C. Chesterman, *Manuel du Dispensaire Tropical*, 4th edn (London: United Society for Christian Literature, Lutterworth Press, 1952).

24.
AA Hygiene 4555 ter, 9 August 1920 letter from the Governor General to the Minister of Colonies.

25.
Annual Public Hygiene Reports 1920–1940, Belgian Congo.

26.
AA RA/CB collection, file 149, 1923 hygiene report by Dr Mouchet on Province Orientale; AA Hygiene 4384, 1927 hygiene report for Stanleyville by Dr Trolli.

27.
AA RA/CB 129, 1922 public hygiene report written by Van Goidtesnoven.

28.
AA Hygiene 4555 ter, folder for 1920–1923, 26 May 1922 letter from the Minister of Colonies to the Governor General.

29.
Splanchnology is the study of visceral organs. AA RA/CB 149, 1923 hygiene report by Dr Mouchet for Province Orientale. My emphasis.

30.
Pieter Huistra and Tinne Claes, ‘Een autopsie is geen dissectie: Hoe Belgische anatomen aan hun lijken kwamen in de late negentiende eeuw’, *unpublished manuscript*, 2015.

31.

*Cerveaux objectifs*, or objective brains, also reads as an odd phrase in the original French.

32.
Introduction to Feierman and Janzen, *op. cit.* (note 7).

33.
Janzen, *op cit.* (note 7), 163.

34.

*Ibid*., 169.

35.

*Ibid*., 169–70.

36.
David Silverman and Brian Torode, *Material Word: Some Theories of Language and Its Limits* (London; Boston, MA: Routledge, 1980), 248.

37.
BMS A/130, ‘Progress and Prospects’ Monthly newsletter of Upoto mission. A story in February 1929 issue by M. Davidson.

38.
John D. Viccars, ‘Witchcraft in Bolobo, Belgian Congo’, *Africa: International Journal of the African Institute*, 19, 3 (1949), 220–9. Similarly, it is observed in parts of Cameroon that the witchcraft substance (*djambe*in Maka, *evu* in Beti, *ewusu* in Duala, or *liemba* in Bakweri) dwells in the stomach or the liver. Peter Geschiere, ‘Chiefs and the Problem of Witchcraft: Varying Patterns in South and West Cameroon’, *The Journal of Legal Pluralism and Unofficial Law*, 28, 37–8 (1996), 307–27.

39.
John H. Weeks, ‘Anthropological Notes on the Bangala of the Upper Congo River. (Part III)’, *The Journal of the Royal Anthropological Institute of Great Britain and Ireland*, 40 (1910), 360–427.

40.
Vansina, *op. cit.* (note 7), 171–2.

41.
E.-H.C. Cambier quoted in *Les Bangalas*, ed. Cyril van Overbergh and Edouard de Jonghe (Brussels: A. D. Witt, 1907), 327.

42.
Edward E. Evans-Pritchard, *Witchcraft, Oracles, and Magic among the Azande* (Oxford Clarendon, 1937); Janzen, *op. cit.* (note 7); Viaene, *op. cit.* (note 8); Viccars, *op. cit.* (note 38).

43.
It could be argued that healing ceremonies involving cutting are also superficial, but they are conceived of as penetrating. They intervene in the body. For more on scarification, see the photos and writings of Emil Torday, one of the most productive anthropologists/ethnologists photographing and discussing scarification in the Belgian Congo. For a relatively recent discussion of scarification, see Megan Vaughan, ‘Scarification in Africa: Re-Reading Colonial Evidence’, *Cultural and Social History*, 4, 3 (2007), 385–400.

44.
Fleeting mentions of surgery can be found in the record, often noting it is rarely done and often ends badly. See, for example, Mary Kingsley, *Travels in West Africa*, 3rd edn (London: Frank Cass, 1965 (1897)), 468.

45.
Nancy Rose Hunt, *A Colonial Lexicon: Of Birth Ritual, Medicalization, and Mobility in the Congo* (Durham, NC: Duke University Press, 1999).

46.
White, *op. cit.* (note 3), 110.

47.
The American Baptist Historical Society archives (ABHS), held at Mercer University, Atlanta, Georgia, contain the holdings of the American Baptist Foreign Missionary Society, which had several medical missions in the Belgian Congo. Collections are organised under the names of individual missionaries. This description is from the papers of Judson King, 7 May 1919 ‘letter to Friends’.

48.
BMS A/128, August 1936 letter from E. W. Price to ‘Prayer Helpers’.

49.
Evans-Pritchard, *op. cit.* (note 42), 490; White, *op. cit.* (note 3). See in particular Ch. 2 of White, *op. cit.* (note 3).

50.
Annual Public Hygiene Reports of Belgian Congo, 1910–40.

51.
Hernias – when an organ is displaced through the wall of a body cavity, usually the intestine through the abdominal wall – can occur spontaneously for unknown reasons, but can also be caused by muscle strain, including strenuous labor.

52.
AA Hygiene 4454, small folder on Red Cross and organisation of Npoko service in 1934.

53.
ABHS Howard Freas, AC Osterholm, and Hjalmar Ostrom papers.

54.
MAE/AA Hygiene 4419. Report: *Voyage d’études au Mayombe* 10 Nov 1924 to 16 Jan 1915 by Dr L. Van Hoof, doctor of the Leopoldville laboratory. When he arrived for epidemiological research, the indigenous people, ‘très méfiants ayant appris l’arrivée d’un médecin du Gouvernement…cachaient leurs malades dans la forêt. Il a fallu plusieurs jours, et *l’appât*des opérations chirurgicales pour que les indigènes des villages voisins amenent leurs malades.’ (My emphasis.).

55.
Janzen, *op. cit.* (note 7), 216.

56.
Terence Ranger observed that surgery was extremely unpopular in Masisi, Tanzania before the Second World War. Terence O. Ranger, ‘Godly Medicine: The Ambiguities of Medical Mission in Southeast Tanzania, 1900–45’, *Social Science and Medicine. Part B Medical Anthropology*, 15, 3 (1981), 261–77.10.1016/0160-7987(81)90052-17031904

57.
BMS Station Reports, A/98 1906 station report from Quibocolo. Quibocolo is technically across the Congo river in Portuguese Angola, but all missions in this region were considered the ‘Congo Mission’, and missionaries regularly crossed the colonial border, thus treating it – or disregarding it – somewhat like indigenous populations did.

58.
Patrick Dixon, *Chibambo Hospital: A Retrospect* (Elisabethville, Belgian Congo: Garenganza Evangelical Mission, 1935), p. 9.

59.
The 1751 English Murder Act specifically laid out the dissection of criminals after their death as part of their punishment. Later reforms to dissection and autopsy practices in the UK could be seen as continued methods to terrorise, exploit and punish marginalised groups. Practices in France differed somewhat. See Anne Carol, *Les Médecins et la mort: XIXe–XXe siècle* (Paris: Aubier, 2004); Ruth Richardson, *Death, Dissection, and the Destitute* (London; New York: Routledge & Kegan Paul, 1987).

60.
Simon Harrison, ‘Skulls and Scientific Collecting in the Victorian Military: Keeping the Enemy Dead in British Frontier Warfare’, *Comparative Studies in Society and History*, 50, 1 (2008), 285–303.

61.
Roger Casement, *Le Rapport Casement: Rapport de R. Casement, consul britannique, sur son voyage dans le Haut-Congo (1903)* (Louvain-la-Neuve: UCL Centre d’histoire de l’Afrique, 1985), 25. Casement also recorded a case where 70 rebels were killed and disemboweled, and their intestines hung over the palisade of the offending village as punishment (11).

62.
Evans-Pritchard, *op. cit.* (note 42), 42.

63.

*Ibid*., 43.

64.
BMS A/130, July 1929 newsletter from M. Davidson, Upoto mission. John Weeks describes this witchcraft substance as something found in the arteries near the liver of the corpse. Viccars describes a similar substance sought in the intestines of witches among the Bolobo, and Vansina mentions in passing the search for this substance post-mortem among the Tio in Middle Congo. John H. Weeks, ‘Anthropological Notes on the Bangala of the Upper Congo River’, *The Journal of the Royal Anthropological Institute of Great Britain and Ireland*, 39 (1909), 416–59; Vansina, *op. cit.* (note 7); Viccars, *op. cit.* (note 38).

65.
BMS A/98, 23 November 1930 letter by a guest to the Quibocolo mission to the Congo Mission News newsletter.

66.
Bernault, ‘Body, Power and Sacrifice’, *op. cit.* (note 7), 213.

67.
David Bunn, ‘The sleep of the brave: graves as sites and signs in the colonial Eastern Cape’, in Paul Landau and Deborah Kaspin (eds), *Images and Empires: Visuality in Colonial and Postcolonial Africa* (Berkeley, CA: University of California Press, 2002), 56–89.

68.
Mahaniah, ‘The Religious Therapeutic Functions of the Cemetery’, *op cit.* (note 7). See also John Middleton, ‘The Concept of “Bewitching” in Lugbara’, *Africa*, 25, 3 (1955), 252–260.

69.
An interesting discussion of how Christian tombstones of Loanga converts come to incorporate indigenous symbolism in a ‘schizophrenic’ fashion is found in James Denbow, ‘Heart and Soul: Glimpses of Ideology and Cosmology in the Iconography of Tombstones from the Loango Coast of Central Africa’, *The Journal of American Folklore*, 112, 445 (1999), 404–23.

70.
Mahaniah, *La Mort dans la pensée Kongo, op cit.* (note 7).

71.
Vansina, *op. cit.* (note 7), 232.

72.
AA Hygiene 4388, 1927 survey of indigenous *infirmiers*.

73.
ABHS Osterholm papers, 1932 report of the medical department of the Vanga mission mentions in passing the autopsy of a Portuguese man at the government’s request, after his suspicious death.

74.
AA RA/CB 150, 1929 Annual Medical Report by Dr Schwetz for Province Orientale.

75.
R. Mouchet, ‘Documents anatomo-pathologiques sur la nosologie de la main-d’oeuvre indigène à Elisabethville de 1915 à 1921’, *Institute Royal Colonial Belge, Bulletin des Séances*, XIV, 2 (1943), 422–52. The fact that men in the prime of their lives are succumbing to dysentery and pneumonia in such high numbers would indicate that research on a ‘local nosological profile’ for the region was meaningless, as mortality was tied to working and living conditions in the mines and not highly dependent on wider epidemiological or biological context.

76.
AA RA/CB 150, 1929 Annual Medical Report by Dr Schwetz for Province Orientale.

77.
AA Hygiene 4393. Note of Dr Moscioni in July 1908 Leopoldville hospital report to the Governor General.

78.
See Maryinez Lyons, *The Colonial Disease: A Social History of Sleeping Sickness in Northern Zaire, 1900–1940* (Cambridge: Cambridge University Press, 1992).

79.
Evans-Pritchard, *op. cit.* (note 42), 490.

80.
The archives of the Ecole de Médecine Tropicale, which is now the Institute of Tropical Medicine in Antwerp, are held at the Institute of Tropical Medicine archives (ITM). The papers of J. Rodhain hold various records of collection and transfer of anatomical materials.

81.
Luise White’s work in particular amasses a huge volume of such stories in several African colonies. White, *op. cit.* (note 3); Patrick Malloy, ‘Research Material and Necromancy: Imagining the Political-Economy of Biomedicine in Colonial Tanganyika’, *International Journal of African Historical Studies*, 47, 3 (2014), 425–43.

82.
AA RA/CB 150,1929 Annual Medical Report by Dr Schwetz for Province Orientale.

83.

*Ibid*.

84.
Janzen, *op. cit.* (note 7), 216.

85.
For a further discussion of cannibalism, political power, punishment and personal relationships in the modern era see Peter Geschiere, *Witchcraft, Intimacy, and Trust: Africa in Comparison* (Chicago, IL: University of Chicago Press, 2013); Peter Geschiere and Janet Roitman, *The Modernity of Witchcraft: Politics and the Occult in Postcolonial Africa* (Charlottesville, VA: University of Virginia Press, 1997).

86.
Simon Harrison, *Dark Trophies: Hunting and the Enemy Body in Modern War* (New York and Oxford: Berghahn Books, 2012), 64.

87.
Laura Franey, ‘Ethnographic Collecting and Travel: Blurring Boundaries, Forming a Discipline’, *Victorian Literature and Culture*, 29, 1 (2001), 219–39 (227–31).

88.
White, *op. cit.* (note 3); Eric Allina, ‘The Zimba, the Portuguese, and Other Cannibals in Late Sixteenth-Century Southeast Africa’, *Journal of Southern African Studies*, 37, 2 (2011), 211–27; Johan Pottier, ‘Rights Violations, Rumour, and Rhetoric: Making Sense of Cannibalism in Mambasa, Ituri (Democratic Republic of Congo)’, *Journal of the Royal Anthropological Institute*, 13, 4 (2007), 825–43.10.1080/03057070.2011.57943322026025

89.
James C. Scott, *Weapons of the Weak: Everyday Forms of Peasant Resistance* (New Haven, CT: Yale University Press, 1985); James C. Scott, *Domination and the Arts of Resistance: Hidden Transcripts* (New Haven, CT: Yale University Press, 1990).

90.
Catherine Mabie, *Congo Cameos* (New York: Judson Press, 1952), 61–2.

